# When traits meet the moment: State and perceived partner motivations interact with approach-avoidance temperament to predict social preferences in daily life

**DOI:** 10.1371/journal.pone.0353754

**Published:** 2026-08-05

**Authors:** Martin Weiß, Karina Zabbarov, Marko Paelecke, Katja Bertsch

**Affiliations:** 1 Department of Psychology I, University of Würzburg, Marcusstr. 9-11, Würzburg, Germany; 2 Department of Psychology V, University of Würzburg, Pleicherwall 1, Würzburg, Germany; University of Reading - Whiteknights Campus: University of Reading, UNITED KINGDOM OF GREAT BRITAIN AND NORTHERN IRELAND

## Abstract

Social interactions are influenced by personality traits such as approach-avoidance motives. However, it remains unclear how traits, states, and interpersonal perceptions interact to shape everyday social experiences – and for whom their interactions are most consequential. Addressing this gap is critical for advancing personality-in-context models and identifying mechanisms underlying deficits in interpersonal functioning. A two-week Ecological Momentary Assessment was conducted with 136 participants (N = 3,991 social interactions), who reported on motivational states, partner perceptions, and interaction preferences. The combined model integrating traits, states, and perceptions best predicted interaction preferences. Higher approach-related traits, states, and perceptions predicted increased interaction preference, whereas avoidance-related states and perceptions (but not avoidance traits) predicted lower interaction preference. Exploratory analyses revealed that deficits in interpersonal functioning were associated with overall lower interaction preference and showed only modest amplifying effects on avoidance-related motives, with underlying slopes remaining directionally consistent across levels of functioning. Short-term lagged associations between motivation and interaction preference were modest and highly time-dependent, with effects dissipating quickly as time between interactions increased. Integrating trait and state perspectives offers valuable insights for understanding social functioning, particularly in individuals at risk for interpersonal difficulties.

## Introduction

Social interactions play a central role in human well-being, contributing to emotional health and overall life satisfaction [1–4]. However, individuals differ markedly in how they engage with, experience, and evaluate these interactions. Theories of social interaction emphasize that interpersonal experiences are shaped by a dynamic interplay between stable personality characteristics and situational factors [5–7]. Broader person–situation frameworks such as the cumulative continuity principle [8] and the TESSERA sequence (Triggering situation, Expectancy, State/State expression, and Reaction;[9]) provide a process-oriented account of how people select, interpret, and modify situations over time, thereby linking momentary states with long-term personality development. The continuity principle highlights that individuals actively create environments consistent with their traits (e.g., a sociable person who repeatedly seeks out group activities and, in doing so, reinforces their own outgoing disposition), whereas TESSERA specifies micro-level feedback loops through which repeated state–situation couplings consolidate or change traits (e.g., a socially anxious person who, on entering a gathering, anticipates rejection, feels tense, and withdraws; across many such episodes these repeated state expressions gradually consolidate into a more avoidant trait pattern). Importantly, both frameworks align with interpersonal theory and whole trait theory [6,10] in proposing that traits manifest in momentary social states, which in turn shape perceptions and reactions during social encounters.

While positive social engagement in social situations generally fosters psychological well-being, individuals with maladaptive personality traits frequently encounter social situations as sources of stress, misunderstanding, or withdrawal, patterns that contribute to chronic interpersonal difficulties and reduced social functioning [11–13]. This contrast mirrors the dual role of approach–avoidance motivational systems: adaptive engagement vs. maladaptive rigidity, depending on the individual’s vulnerabilities and interpersonal skill.

One concept for understanding individual differences in social interactions is the approach-avoidance temperament [14]. This conceptualization posits that behavior is guided by two fundamental motivations at the trait-level: the tendency to approach rewarding stimuli and the tendency to avoid threatening or aversive situations. These motivational tendencies have been associated with social outcomes such as levels of social engagement, relationship satisfaction, and susceptibility to interpersonal difficulties [15,16]. Within broader models of personality, approach- avoidance temperament has been shown to overlap substantially with, yet remain distinct from, Big Five traits (e.g., extraversion, neuroticism; see 14) and it can be meaningfully situated alongside interpersonal circumplex (agency–communion) dimensions that are widely used in EMA research to describe interpersonal behavior in daily life [17–19]. On this circumplex, the agency dimension captures the extent to which behavior is dominant versus submissive, whereas the communion dimension captures the extent to which it is warm versus cold or hostile. Approach motivation maps most closely onto agentic and communal (i.e., engaged, warm) behavior, whereas avoidance motivation maps onto cold and withdrawn behavior, which is why the agency-communion framework provides a useful descriptive language for the motivational states examined here. While these motivations operate across the full spectrum of functioning in the general population, extreme or inflexible patterns can signal maladaptive personality organization, extending even into clinical personality disorders. For example, persistent social avoidance may lead to chronic isolation, whereas dysregulated approach can precipitate interpersonal conflicts and boundary violations [12,20]. These directional temperaments, however, do not tell us how skillfully people pursue their social goals. This is where the construct of interpersonal functioning is relevant, defined as the capacity to initiate, maintain, and repair relationships [11]. It indexes the skill and flexibility with which social behavior is enacted and is therefore conceptually distinct from approach–avoidance temperament. Deficits in interpersonal functioning can magnify motivational effects by heightening threat perception and narrowing behavioral repertoires, a process the TESSERA sequence characterizes as repeated maladaptive loops that ultimately consolidate into trait-level dysfunction.

However, trait-level motivations such as approach and avoidance temperament alone offer an incomplete picture. Social interactions are inherently dynamic, shaped not only by stable traits but also by a range of situational factors – both external context cues and internal motivational states – as well as real-time perceptions of others [6,21–23]. Complementing TESSERA, the vulnerability–stress–adaptation model [24,25] shows that enduring vulnerabilities (e.g., avoidance temperament) interact with situational stressors to predict relationship processes, underscoring the value of studying cross‑level interactions. For instance, even individuals high in avoidance temperament may display situational variability, i.e., engaging socially when contexts feel safe or rewarding. Similarly, perceptions of an interaction partner’s openness or avoidance can significantly shape the subjective evaluation of an encounter, particularly in individuals prone to interpersonal sensitivity or maladaptive interpretations [26]. EMA research in daily life further shows that people’s perceptions of interaction partners (e.g., warmth, responsiveness, agency-/communion-related behaviors) are among the strongest moment-to-moment predictors of their affective states and interpersonal behaviors [27–29]. Among such partner cues, the perceived physical attractiveness of an interaction partner is a particularly salient reward signal that has been linked to heightened approach motivation (e.g., [30]) and more positive social evaluations [31,32]. From an approach–avoidance perspective, attractiveness functions as a positively valenced incentive that can amplify approach-related responding independently of a partner’s perceived motivation [33,34]. We therefore included perceived partner attractiveness as a perception-level covariate in all models to ensure that the effects of motivational appraisals were not confounded with this basic reward cue.

In recent years, research has increasingly recognized the importance of integrating state-level motivations with trait models to better understand personality in situational context [5,35–38]. Ecological Momentary Assessment (EMA) has proven especially valuable in capturing these dynamic processes, allowing for real-time assessment of social behavior, affect, and motivation in naturalistic settings [39]. EMA studies have demonstrated that fluctuations in social approach and avoidance states are closely linked to daily well-being [40,41]. Building on our prior discussion of how rigid or extreme approach–avoidance temperament patterns constitute maladaptive personality functioning, these EMA investigations reveal that individuals at the maladaptive end of the spectrum exhibit heightened interpersonal reactivity, manifesting as stronger perceptions of rejection and greater fluctuations in desire for social contact [42–44]. Moreover, findings suggest that both one’s own motivational states and the perceived motives of interaction partners play critical roles in shaping evaluation of social encounters [45–47].

In consequence, a well-established line of work in personality and social psychology has emphasized the need to integrate state-level variability with trait-level perspectives to better predict behavior and subjective experiences [5,36,38]. Here, we use approach and avoidance temperament to refer to stable, trait-level approach-avoidance motivation, state-level approach and avoidance motivations to refer to how inclined individuals feel to engage or withdraw at any given moment, and perceived partner approach and avoidance motivations to capture how one appraises an interaction partner’s openness or avoidant stance. While many situational factors (e.g., setting, stressors, group norms) can shape social encounters, we focus on these three components because they directly map onto approach–avoidance dynamics and have been shown to drive social behavior and evaluation [48]. Crucially, our theoretical interest lies not only in their separate effects but in how they combine, i.e., interact, to determine whether people want to engage in a given social encounter. Despite abundant work on each component in isolation, there is no study that has simultaneously considered trait- and state-level approach-avoidance motivation, as well as perceived partner approach-avoidance motivation to predict subjective social outcomes. This layered perspective reflects how processes operate across levels: traits predispose, states fluctuate in context, and partner perceptions shape the meaning of those states.

**Fig 1:**
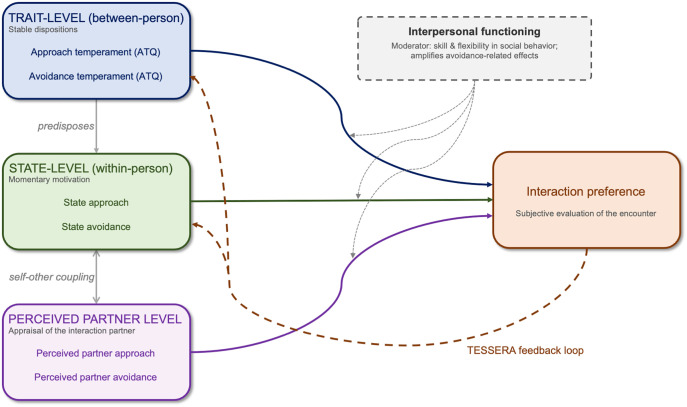
An integrative approach–avoidance model of social interaction preference. Momentary interaction preference is predicted by three levels: stable traits (approach/avoidance temperament; ATQ), within-person states (state approach/avoidance), and partner appraisals (perceived partner approach/avoidance). Traits predispose states, which couple with partner perceptions during an encounter. Interpersonal functioning (LPFS-BF Interpersonal) moderates these paths, especially avoidance-related effects. Solid colored arrows = predictive paths estimated in the multilevel models; grey dashed arrows = moderation; the orange dashed arrow = the theoretically expected TESSERA feedback loop (consolidation of states into traits over time), which is conceptual and not tested here.

To address this gap, we conducted a two-week EMA study investigating how the interaction between trait- and state-level approach-avoidance motivations, and perceived partner motivations predicts the subjective evaluation of social interactions in daily life. Given that prior EMA research has modeled associations between motivational states and social outcomes predominantly using linear mixed-effects models, with consistently monotonic effects and no evidence for nonlinearities [49–51], and considering the restricted variance of trait approach in our sample, we tested linear hypotheses. We hypothesized that considering these components simultaneously will better explain interaction preferences than examining traits or states in isolation. For the individual components, we hypothesized that higher trait approach temperament, higher state approach and higher perceived approach motivation predict an increase in interaction preference, whereas higher trait avoidance temperament, higher state avoidance and higher perceived avoidance motivation predict a decrease in interaction preference. Additionally, this study aims to explore how deficits in interpersonal functioning may moderate these processes, with the expectation that deficits in interpersonal functioning would amplify avoidance-related processes, offering a more differentiated understanding of personality influences in everyday social experiences. **Fig 1** provides a conceptual overview of the tested relations.

## Method

### Transparency and openness

We report how we determined our sample size, all data exclusions, all manipulations, and all measures in the study, and we follow JARS [52]. All data, analysis code, and research materials are available at https://doi.org/10.17605/osf.io/hmw4k. Data were analyzed using R, version 4.4.1. This study's design and its analysis were preregistered at https://doi.org/10.17605/osf.io/9ckx6.

### Participants

Between January and April 2025, 181 individuals started the EMA phase of the study (see below for details). We included participants who were at least 18 years old and fluent in German. As pre-registered, we included only participants in our analyses who took part in at least 50% (≥ 28) of the 56 possible prompts without failing the attention checks and who indicated in at least 25% (≥ 14) of all possible prompts that they had a social interaction since the previous prompt. From the 181 persons who installed the m-Path smartphone application (m-path.io), five did not answer a single prompt, 20 participants failed exactly one exclusion criterion and 20 failed both criteria, which resulted in a final data set of 136 individuals (mean age = 27.85, *SD* = 7.32 years, range 19−64; 104 women, 30 men, 1 other, 1 did not wish to indicate, mean subjective socioeconomic status = 5.64, SD = 1.47). These participants responded on average to 49.6/56 prompts (i.e., 89% compliance; *SD* = 6.98, range = 28−56). Of these, 29.3 prompts on average (*SD* = 8.58) targeted direct personal social interactions (*n* = 3991 social prompts). Because the study targeted recent, in-person social interactions, we did not consider 1,969 interactions that had occurred more than 60 minutes before the prompt or 780 interactions that had taken place virtually. We preregistered 135 participants and calculated the sample size using the *ema.powercurvefunction* from the *R*-package EMATools [53]. The resulting sample size was based on an intra-class coefficient (ICC) of 0.37 [23], an expected mean completion rate of ≥ 75% and 90% power. Under these assumptions, the targeted sample provided adequate statistical power for our primary multilevel analyses. To further contextualize this decision, comparable EMA studies that explicitly separated within- and between-person effects have used similar sizes ([23] N = 130, [42] N = 113, [54] N = 114, [55] N = 129, [56] N = 120). Participants earned €0.25 for each prompt answered and an additional €6 for completing the intake survey, which included demographic information, trait questionnaires, and an explanation of the study procedure, resulting in a maximum total fee of €20. All participants gave written informed consent in accordance with the Declaration of Helsinki before they participated in the study. The protocol was approved by the local ethics committee (approval: GZEK 2024−69). Before providing consent, all participants were given a participant information overview describing the purpose, procedure, data handling, and the voluntary and confidential nature of participation, and they were debriefed about the aims of the study at the end of the EMA period.

### Procedure

First, participants completed a series of online questionnaires, collecting detailed data on socio-demographic characteristics (including the MacArthur Scale as a measure of subjective social status; [57]), as well as several trait measures (see below). Second, participants entered a 14-day EMA phase using the m-Path app. During this period, participants received four daily prompts at randomized times between 10–11 a.m., 1–2 p.m., 4–5 p.m., and between 7–8 p.m., directing them to complete a short questionnaire tailored to their recent experiences. Reminder notifications were sent 30 minutes after each prompt, and participants had a two-hour response window, after which the survey expired. If a social interaction has occurred since the previous prompt, participants evaluated the most recent interaction, reflecting on its quality and features of the interaction partner(s), their approach and avoidance state as well as the perceived approach and avoidance state of their interaction partner(s). If no social interaction took place, they completed an alternative questionnaire of equal length to avoid response biases. This version inquired about their current emotions, and whether they would have liked to interact with others later that day. The list of EMA items can be found in the OSF repository (https://doi.org/10.17605/osf.io/hmw4k).

## Measures

### Trait questionnaires

Participants were asked to answer the following trait measures: the German version of the Approach-Avoidance Temperament Questionnaire (ATQ; [58]; see also [59]; Cronbach’s α in the present sample = .78 for approach and  .88 for avoidance) and the Level of Personality Functioning Scale, Brief Form 2.0 (LPFS-BF; [60]). In the current manuscript, we focused exclusively on the *interpersonal* subscale (LPFS-BF Interpersonal; Cronbach’s α = .75 in the present sample). In addition, we assessed the modified version of the Personality Inventory for DSM-5 – Brief Form Plus [61] and a friendship goals questionnaire [62] for exploratory purposes. Descriptive intercorrelations among these additional trait measures are reported in Figure S1 of the Supplementary Materials in S1 File. The order of the trait measures was randomized across participants.

### EMA survey

For the analysis presented in this manuscript, the following EMA items were relevant. First, participants were asked whether they had engaged in a social interaction within the past 60 minutes, with response options: “Yes, the interaction took place in direct personal contact,” “Yes, the interaction took place online/via phone,” or “No.” This procedure was implemented a priori to ensure that all primary EMA reports referred to in-person encounters, given that such interactions provide richer affiliative and motivational cues than technology-mediated exchanges (e.g., [63]). Subsequent items were administered and recorded only if participants indicated that a direct, in-person social interaction had occurred. Alternative items, which were not pertinent to this analysis, are available in the OSF repository. Participants were then asked whether they interacted with one or multiple individuals, and to specify the nature of their interaction partner(s) – choosing from romantic partner, colleague(s), family member(s), friend(s), acquaintance(s), or stranger(s). Additionally, they rated the physical attractiveness of their interaction partner(s) using a slider ranging from 0 (“not at all”) to 100 (“extremely”).

The following items, all rated on sliders from 0 to 100, focused specifically on the social interaction. To assess state approach, participants rated how motivated and interested they felt using two items (“I did have a lot of motivation in this interaction.” and “I was interested in my interaction partner.”). Similarly, perceived approach by the interaction partner was evaluated by asking how motivated and interested the partner appeared to them (“I think my interaction partner had a lot of motivation in this interaction.” and “I think my interaction partner was interested in me.”). State avoidance was measured with two items assessing how much participants wished to avoid the interaction and how much they would have preferred to be alone (“Actually, I wanted to avoid the conversation or the person I was talking to.” and “I wanted to be alone instead of interacting with the person.”). For perceived avoidance by the partner, participants rated the extent to which they believed these two aspects applied to their interaction partner (“I think my interaction partner wanted to avoid the conversation/me.” and “I think my interaction partner wanted to be alone instead of interacting with me.”).

To account for affective valence, we included state positive and negative affect [64] as control variables in all main models. We selected the PANAS items (“enthusiastic,” “happy,” “determined” for positive affect; “worried,” “nervous,” “anxious” for negative affect) based on their conceptual similarity and empirical association with approach and avoidance temperament (see [58]). Including these affect indicators allows us to isolate the directional, motivational aspects of approach and avoidance from mood or arousal. Finally, interaction preference was captured by asking participants how much they liked interacting with the person (“I did like to interact with this person.”; 0 = “not at all”; 100 = “extremely”). Across prompts, all multi-item EMA composites showed high internal consistency in the present sample (state approach, Cronbach’s α = .83; perceived partner approach, α = .85; state avoidance, α = .87; perceived partner avoidance, α = .86; state positive affect, α = .87; state negative affect, α = .85; see Table 1). Interaction preference and partner attractiveness were assessed with single items and therefore have no internal-consistency estimate.

**Table 1:** Descriptive statistics for between-person (trait) and within-person (state) variables.

Variable	α	n	M	SD	Min	Max
*Between-Person Variables*						
ATQ Approach	.78	136	5.02	0.87	2.83	6.83
ATQ Avoidance	.88	136	4.30	1.35	1.00	7.00
LPFS-BF Interpersonal	.75	136	1.74	0.52	1.00	3.00
*Within-Person Variables*						
Attractiveness	–	3,991	39.33	38.30	0.00	100.00
State positive affect	.87	3,991	60.09	22.52	0.00	100.00
State negative affect	.85	3,991	14.28	17.63	0.00	100.00
State approach	.83	3,991	70.66	22.76	0.00	100.00
Perceived state approach	.85	3,991	71.09	22.67	0.00	100.00
State avoidance	.87	3,991	22.59	25.79	0.00	100.00
Perceived state avoidance	.86	3,991	17.77	20.87	0.00	100.00
Interaction preference	–	3,991	74.84	23.66	0.00	100.00

*Note*. M = Mean; SD = Standard Deviation. Between-person variables reflect aggregated trait-level data (n = 136 participants). Within-person variables reflect prompt-level assessments across interactions (n = 3,991 observations). ATQ = Approach-Avoidance Temperament Questionnaire; LPFS-BF = Level of Personality Functioning Scale – Brief Form.

### Analyses

To test our pre-registered hypotheses, we used linear mixed-effects models to examine the predictors of interaction preference. In all linear mixed effects models, continuous within-person variables were person-centered (centered-within [cw]) and continuous between-person variables were grand mean-centered (centered-between [cb]; [65]). All results that were not preregistered were labelled as exploratory in the results section. All models accounted for the nested structure of the EMA data (Level 1: interactions; Level 2: persons) using random intercepts for participants. As pre-registered state positive affect, state negative affect, and physical attractiveness of the interaction partner were included as control variables.

Model selection was based on the Akaike Information Criterion (AIC), which balances model fit and parsimony in multilevel designs [66]. For completeness, we also report the Bayesian Information Criterion (BIC) in Supplementary Tables. In addition to information criteria, marginal and conditional R² values [67] were reported to convey explained variance; this constitutes a minor deviation from the preregistration, added for transparency and interpretability.

### Confirmatory models

We estimated a series of five confirmatory multilevel models to examine how motivational traits and states jointly predict social interaction preferences. The trait-only model (M1) included approach and avoidance temperament as between-person predictors to test whether stable motivational tendencies relate to average interaction preference. The state-only model (M2) focused on within-person variability, examining whether momentary state approach and state avoidance motivation during an interaction predicted concurrent interaction preferences. The perceived state-only model (M3) included perceived partner approach and perceived partner avoidance motivation as within-person predictors to capture how perceptions of a partner’s motivation shape one’s own interaction experience. The combined model (M4) integrated both levels by including trait-level approach and avoidance temperament together with state and perceived states, as well as their theoretically corresponding interactions (trait approach × state approach × perceived partner approach; trait avoidance × state avoidance × perceived partner avoidance). This model thus tested how dispositional motivation moderates the impact of situational dynamics on social preference. The maladaptive model (M5) extended the trait-only model by including LPFS-BF Interpersonal functioning and its interactions with trait approach and avoidance temperament to test whether deficits in interpersonal functioning amplify or buffer dispositional effects on social evaluation.

### Robustness and sensitivity analyses

We examined whether relationship type accounted for additional variance in interaction preferences by adding it as a fixed effect to the combined model (M4). Adding relationship type as a fixed effect did not improve model fit; both information criteria favored the simpler model (AIC = 32310 vs. 32293; BIC = 32467 vs. 32419). We therefore retained the more parsimonious specification. Consequently, relationship context did not explain additional variance beyond motivational and affective predictors, and we retained the more parsimonious specification.

To ensure that between-person effects were appropriately captured by baseline trait measures, we conducted a sensitivity analysis comparing models that used aggregated EMA means of state approach and avoidance as trait proxies to those using baseline temperament scores (M1). This approach allowed us to test whether the observed between-person effects reflected stable motivational tendencies rather than statistical artifacts of aggregation. The results (see Supplementary Table S1 in S1 File) showed nearly identical effect patterns, with aggregated EMA means providing slightly better model fit, but not altering the substantive conclusions. Thus, baseline trait measures appear to validly represent stable motivational dispositions in this context.

Finally, to evaluate whether controlling for affect might remove substantive variance from state approach and avoidance motivation, we re-estimated the state (M2) and combined (M4) models without PANAS controls (positive and negative affect). Model comparisons (see Table S2 S1 File) showed that including affect significantly improved model fit (all *p*s < .001) but did not alter the magnitude or direction of motivational predictors. This suggests that approach–avoidance effects are not reducible to affective valence, supporting their interpretation as directional motivational processes rather than mood-related artifacts.

### Exploratory models

An exploratory mixed-effects model (M6) was tested to examine whether deficits in interpersonal functioning (LPFS-BF Interpersonal) further moderated the relationships between trait temperament, state-level motivations, and interaction preferences. This model included four-way interactions between interpersonal functioning (LPFS-BF Interpersonal), trait approach and avoidance temperament, and state-level (perceived) approach and avoidance. The same covariates (state positive and negative affect, attractiveness) and random intercept structure as in the main combined model were retained.

Next, to examine short-term within-person carryover effects, we estimated lag-1 multilevel models in two directions. In the forward direction (M7), next interaction preference (*t + 1*) was predicted from motivational states at *t* (state approach, state avoidance, perceived partner approach, perceived partner avoidance), with the log-transformed inter-interaction time gap (Δtime; z-standardized) included as a covariate and as an interaction term with each state to model temporal decay. In a robustness check, we included interaction preference at *t* as an autoregressive control; however, this addition did not improve model fit (Δχ²(1) = 0.91, *p* = .341), and the simpler model without this term was retained.

In the reverse direction, the next state approach (M8) and next state avoidance (M9) were predicted in two separate models from lagged interaction preference (person-mean centered, z-standardized), controlling for the current state (autoregressive stability), Δtime, and their interactions. All predictors were person-mean centered and standardized; all models included fixed effects of trait approach and avoidance temperament (ATQ) and random intercepts for participants to account for between-person differences.

In an additional exploratory model, we examined whether approach and avoidance temperament moderated the momentary effects of state and perceived partner motivation on interaction preference by including all trait × state × perceived-state interactions. Full model specifications and complete results are reported in the Supplement.

## Results

Descriptive statistics for all relevant variables are presented in Table 1. Participants reported mainly on interactions with family members (25%), followed by partners (24%), friends (20%), colleagues (14%) and strangers (8%). Overall, participants reported moderate levels of trait approach and avoidance temperament, with relatively low levels of deficits in interpersonal functioning. State-level approach motivation and perceived partner approach were generally high across interactions, whereas (perceived) avoidance motivations were lower. The mean rating for interaction preference was relatively high, indicating that participants typically evaluated their social interactions positively. All EMA-based affective and motivational composites demonstrated high internal reliability across prompts (Cronbach’s α = .83–.87; see **Table 1**), supporting that the brief multi-item indices provided reliable assessments of momentary affect and motivation during social interactions. Across all EMA variables, skewness values ranged from –1.1 to 1.6 and kurtosis from –1.4 to 2.5, indicating only mild deviations from normality. Approach-related variables and positive affect showed slight negative skew, reflecting ceiling tendencies, whereas avoidance-related variables and negative affect displayed moderate positive skew, indicating that these experiences occurred less frequently but were occasionally pronounced.

**Fig 2:**
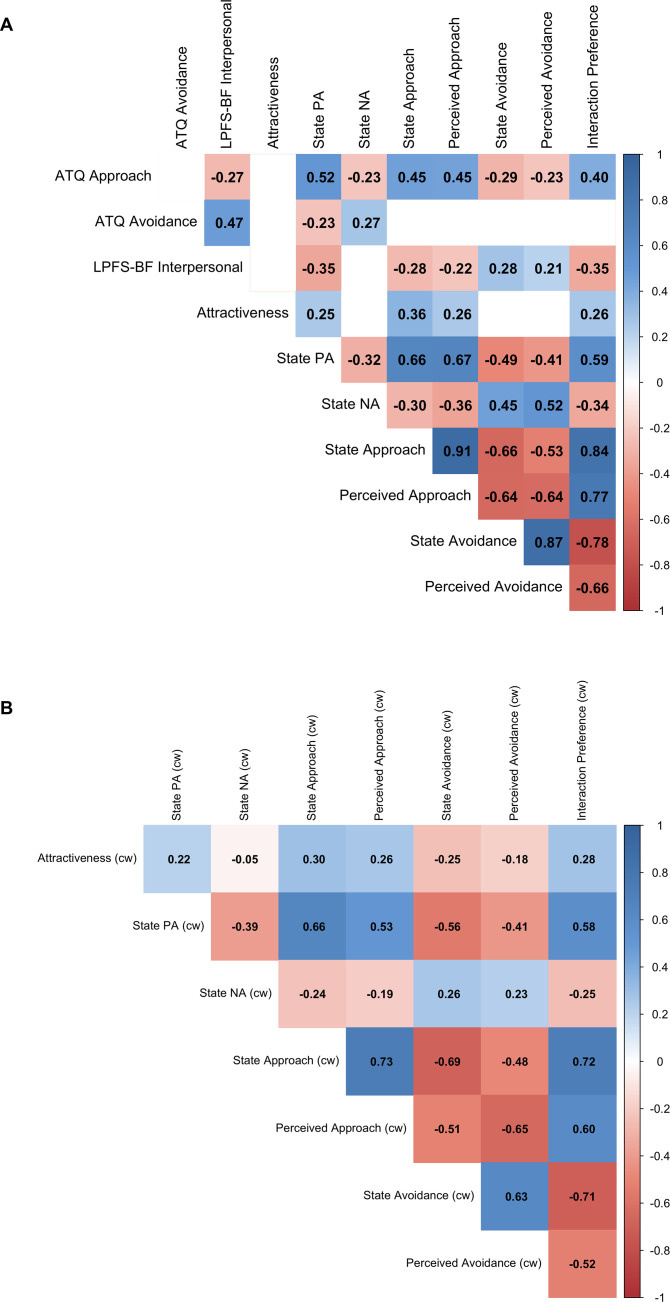
Correlation matrices at (A) the between-person level (person-mean aggregated EMA variables, along with baseline traits) and (B) the within-person level (person-centered EMA variables). Coefficients are shown for significant associations (*p* < .05); non-significant cells are blank.

To examine the conceptual distinctiveness and convergence of motivational variables, we computed correlation matrices separately for between-person (person-mean aggregated) and within-person (centered) levels (**Fig 2**). At the between-person level (**Fig 2A**), all motivational indicators were strongly interrelated. State approach correlated positively with perceived partner approach (*r* = .91) and negatively with both state (*r* = –.66) and perceived partner avoidance (*r* = –.53). Similar cross-associations emerged for the affective control variables, with state PA positively associated with state approach (*r* = .66) and negatively with state NA (*r* = –.32). Baseline trait approach temperament correlated moderately with both state and perceived approach (*r*s = .45). Deficits in interpersonal functioning (LPFS-BF Interpersonal) showed small-to-moderate negative correlations with approach temperament (*r* = –.27) and positive correlations with avoidance temperament (*r* = .47), consistent with its maladaptive interpersonal characterization. At the within-person level (**Fig 2B**), correlations were weaker but followed the same pattern: State approach was positively associated with perceived partner approach (*r* = .73) and negatively with both state (*r* = –.69) and perceived partner avoidance (*r* = –.48). Overall, the results indicate consistent alignment between self- and partner-directed motivation, while the lower within-person correlations confirm that these constructs retain situationally specific variance beyond shared valence or perception biases.

### Are traits, states, and perceived states better alone or together?

As shown in Table 2, the combined model (AIC = 32293) provided the best overall fit, compared to models focused solely on traits (AIC = 34321), states (AIC = 32394), perceived states (AIC = 33516), and the one including maladaptive traits (AIC = 34313).

**Table 2:** Model comparison using AIC, BIC and R² values.

Model	AIC	BIC	Marginal R²	Conditional R²
M1: Trait Only	34321	34371	0.325	0.482
M2: State Only	32394	32444	0.489	0.687
M3: Perceived State Only	33516	33566	0.387	0.582
**M4**: **Combined**	**32293**	**32419**	**0.531**	**0.695**
M5: Maladaptive	34313	34382	0.341	0.484
M6: Exploratory	32277	32498	0.548	0.698

*Note*. AIC = Akaike Information Criterion; BIC = Bayesian information criterion; Marginal R² reflects variance explained by fixed effects; Conditional R² reflects variance explained by both fixed and random effects. The combined model demonstrated the best overall fit (lowest AIC) while explaining a substantial proportion of variance.

The combined model also accounted for the largest proportion of variance in interaction preferences (Marginal R² = .531) compared to the trait-only (R² = .325), state-only (R² = .489), perceived state-only (R² = .387), and maladaptive trait models (R² = .341). The exploratory model yielded a slightly lower AIC than the combined model (32277 vs. 32293) and a somewhat higher marginal R² (.548), but also a higher BIC (32498 vs. 32419), indicating improved fit at the cost of additional complexity.

A detailed summary of the confirmatory models’ results is presented in **Table 3**. For the combined model, results showed that trait approach temperament was positively associated with interaction preference (*B* = 4.32, 95% CI [2.60, 6.04], *p* < .001). Both state approach motivation (*B* = 6.28, 95% CI [5.47, 7.09], *p* < .001) and perceived partner state approach (*B* = 2.05, 95% CI [1.31, 2.79], *p* < .001) also predicted higher interaction preference. In contrast, state avoidance (*B* = –7.37, 95% CI [–8.06, –6.69], *p* < .001) and perceived partner state avoidance (*B* = –0.78, 95% CI [–1.46, –0.10], *p* = .024) were associated with lower interaction preference. Trait avoidance was not a significant predictor (*p* = .307).

**Table 3:** Fixed effects estimates, confidence intervals, and significance levels for mixed-effects models predicting interaction preferences.

Predictor	M1: Traits only	M2: States only	M3: Perceived states	M4: Combined	M5: Maladaptive	M6: Exploratory
Intercept	**74.26 [72.57, 75.96]**	**74.24 [72.39, 76.09]**	**74.25 [72.40, 76.10]**	**74.52 [72.81, 76.24]**	**75.07 [73.21, 76.94]**	**75.37 [73.48, 77.26]**
Approach temp.	**4.36 [2.66, 6.06]**			**4.32 [2.60, 6.04]**	**3.47 [1.74, 5.20]**	**3.33 [1.59, 5.08]**
Avoidance temp.	−0.92 [−2.62, 0.78]			−0.88 [−2.59, 0.83]	0.29 [−1.59, 2.17]	0.43 [−1.47, 2.33]
Interpersonal funct.					**−2.69 [−4.73, −0.66]**	**−3.06 [−5.11, −1.00]**
State positive affect	**11.26 [10.67, 11.85]**	**2.39 [1.81, 2.98]**	**6.96 [6.35, 7.56]**	**2.18 [1.60, 2.77]**	**11.26 [10.67, 11.85]**	**2.10 [1.51, 2.69]**
State negative affect	**−0.81 [−1.39, −0.24]**	−0.44 [−0.89, 0.01]	**−0.57 [−1.09, −0.04]**	−0.42 [−0.86, 0.03]	**−0.81 [−1.39, −0.24]**	**−0.49 [−0.94, −0.04]**
Partner attractiveness	**3.49 [2.94, 4.03]**	**1.14 [0.70, 1.57]**	**2.20 [1.70, 2.70]**	**1.05 [0.62, 1.48]**	**3.49 [2.94, 4.03]**	**1.03 [0.59, 1.46]**
State approach		**7.92 [7.27, 8.57]**		**6.28 [5.47, 7.09]**		**6.12 [5.30, 6.94]**
State avoidance		**−7.82 [−8.40, −7.24]**		**−7.37 [−8.06, −6.69]**		**−7.04 [−7.81, −6.26]**
Perceived partner approach			**5.76 [5.07, 6.45]**	**2.05 [1.31, 2.79]**		**2.16 [1.40, 2.91]**
Perceived partner avoidance			**−3.96 [−4.59, −3.32]**	**−0.78 [−1.46, −0.10]**		**−0.83 [−1.61, −0.05]**
Approach temp. × state approach				0.45 [−0.11, 1.01]		**0.67 [0.07, 1.27]**
Approach temp. × perceived approach				−0.11 [−0.67, 0.46]		−0.32 [−0.91, 0.28]
State × perceived approach				**−0.45 [−0.83, −0.07]**		**−0.42 [−0.81, −0.04]**
Avoidance temp. × state avoidance				−0.48 [−0.98, 0.01]		−0.45 [−1.09, 0.18]
Avoidance temp. × perceived avoidance				−0.06 [−0.56, 0.45]		−0.06 [−0.70, 0.58]
State × perceived avoidance				0.09 [−0.27, 0.45]		−0.04 [−0.47, 0.39]
Approach temp. × state × perceived approach				0.02 [−0.29, 0.34]		0.16 [−0.17, 0.49]
Avoidance temp. × state × perceived avoidance				−0.08 [−0.38, 0.21]		−0.28 [−0.67, 0.11]
Interpersonal funct. × approach temp.					−0.04 [−1.66, 1.58]	−0.20 [−1.84, 1.45]
Interpersonal funct. × avoidance temp.					**−1.73 [−3.38, −0.08]**	**−1.75 [−3.42, −0.09]**
Interpersonal funct. × state approach						0.47 [−0.29, 1.22]
Interpersonal funct. × perceived approach						**−1.17 [−1.91, −0.42]**
Interpersonal funct. × state avoidance						0.14 [−0.63, 0.91]
Interpersonal funct. × perceived avoidance						−0.69 [−1.46, 0.08]
Interpersonal funct. × approach temp. × state approach						−0.40 [−0.96, 0.16]
Interpersonal funct. × approach temp. × perceived approach						0.37 [−0.21, 0.94]
Interpersonal funct. × state × perceived approach						0.28 [−0.08, 0.65]
Interpersonal funct. × avoidance temp. × state avoidance						**−0.54 [−1.06, −0.02]**
Interpersonal funct. × avoidance temp. × perceived avoidance						0.11 [−0.44, 0.66]
Interpersonal funct. × state × perceived avoidance						0.22 [−0.22, 0.66]
Interpersonal funct. × approach temp. × state × perceived approach						0.25 [−0.05, 0.55]
Interpersonal funct. × avoidance temp. × state × perceived avoidance						0.10 [−0.21, 0.42]

*Note*. Estimates are unstandardized coefficients with 95% confidence intervals in brackets. Approach Temp. = Approach Temperament; Avoidance Temp. = Avoidance Temperament; Interpersonal funct. = Interpersonal Functioning Blank cells indicate that the predictor or interaction term was not included in the respective model. All bold marked cells are significant at an alpha level of *p* < .05.

**Fig 3:**
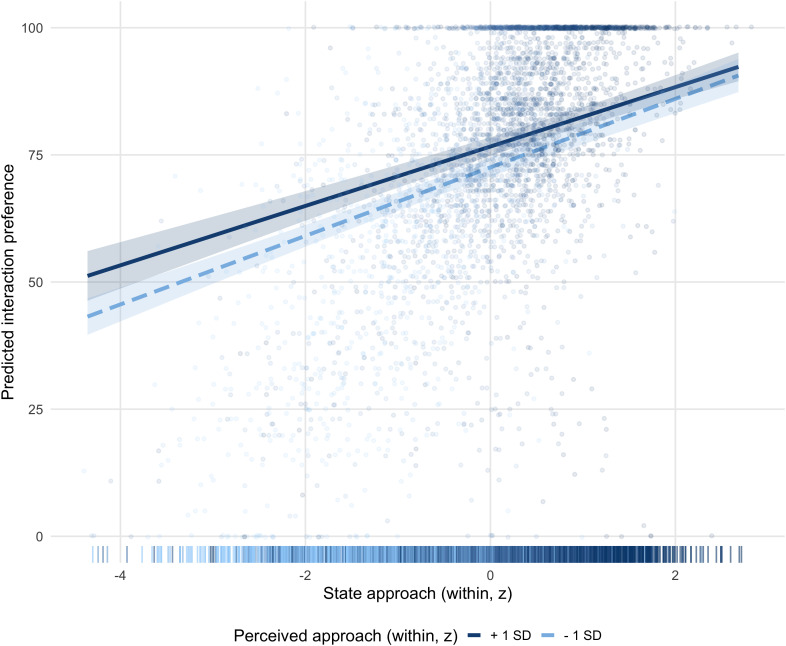
Interaction between state approach motivation and perceived partner approach predicting interaction preference. Shaded areas represent 95% confidence intervals.

A small but significant interaction emerged between state approach and perceived state approach (*B* = –0.45, 95% CI [–0.83, –0.07], *p* = .020, **Fig 3**), suggesting a diminishing return: The effect of state approach on interaction preference was slightly weaker when perceived partner state approach was already high. Simple-slopes analyses confirmed that the slope of state approach remained strongly positive at both low levels of perceived partner approach (–1 SD: B = 6.74, *p* < .001) and high levels (+1 SD: B = 5.84, *p* < .001). No other two- or three-way interactions were statistically significant (all values of *p* ≥ .055).

Of the covariates, positive affect (*B* = 2.18, 95% CI [1.60, 2.77], *p* < .001) and partner attractiveness (*B* = 1.05, 95% CI [0.62, 1.48], *p* < .001) were both associated with greater interaction preference, whereas negative affect was not a significant predictor (*p* = .069).

In the maladaptive trait model including deficits in interpersonal functioning, higher levels of such deficits (LPFS-BF Interpersonal) were significantly associated with lower interaction preferences (*B* = –2.69, 95% CI [–4.73, –0.66], *p* = .009). Additionally, a significant interaction was found between interpersonal functioning (LPFS-BF Interpersonal) and trait avoidance temperament (*B* = –1.73, 95% CI [–3.38, –0.08], *p* = .040), indicating that trait avoidance was more negatively associated with interaction preferences among individuals with higher levels of deficits in interpersonal functioning. However, simple-slopes analyses showed that the effect of trait avoidance was not significant at either low levels of interpersonal dysfunction (–1 SD: B = 2.00, *p* = .092) or high levels (+1 SD: B = –1.40, *p* = .293). The Johnson–Neyman interval further indicated that the slope of trait avoidance temperament was never significant across the observed range of deficits in interpersonal functioning. Thus, although the interaction term reached significance, the simple-slopes pattern suggests that trait avoidance temperament did not show reliably stronger negative effects at higher levels of deficits in interpersonal functioning. No significant interaction emerged between interpersonal functioning and trait approach temperament (*p* = .961).

### Exploratory moderation analyses

Consistent with the combined models tested before, higher trait approach temperament, greater state approach motivation, and perceiving partners as more approach-oriented predicted increased interaction preference (all *p*s < .001). Conversely, deficits in interpersonal functioning (LPFS-BF Interpersonal), state avoidance and perceiving partners as avoidant predicted lower interaction preference (all *p*s ≤ .037).

**Fig 4:**
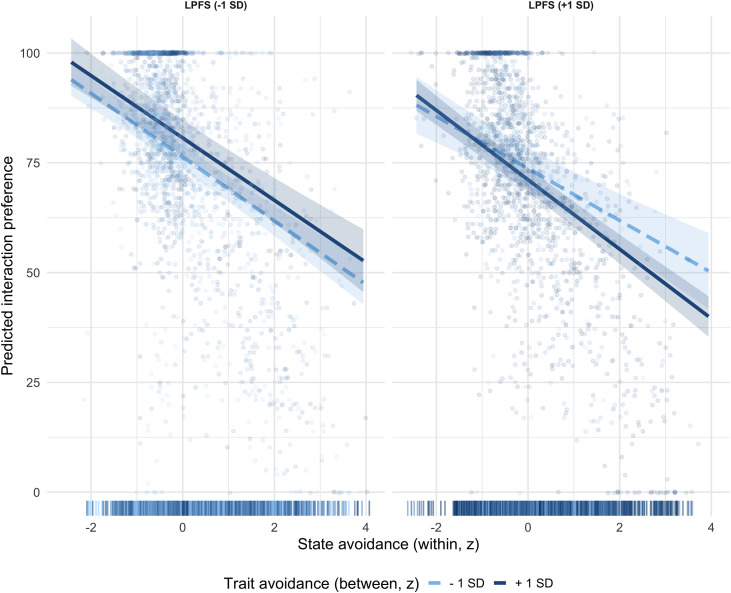
Three-way interaction between maladaptive interpersonal functioning (LPFS-BF Interpersonal), avoidance temperament, and momentary state avoidance predicting interaction preference. Shaded areas represent 95% confidence intervals.

Beyond these, the exploratory model revealed additional interactions in how maladaptive interpersonal functioning, trait approach-avoidance temperament, and state-level motivation interact to shape social preferences. Importantly, deficits in interpersonal functioning (LPFS-BF Interpersonal) amplified the negative impact of trait avoidance temperament on social evaluations, as indicated by an interaction between LPFS-BF Interpersonal and trait avoidance temperament (*B* = –1.75, 95% CI [–3.42, –0.09], *p* = .039). Furthermore, individuals high in trait avoidance temperament evaluated interactions more negatively in moments characterized by heightened state avoidance when coupled with greater deficits in interpersonal functioning (LPFS-BF Interpersonal), reflected in a significant three-way interaction (*B* = –0.54, 95% CI [–1.06, –0.02], *p* = .043, **Fig 4**). However, simple-slopes analyses showed that state avoidance was a strong negative predictor of interaction preference at all levels of the moderators, regardless of trait avoidance or interpersonal dysfunction. When interpersonal dysfunction was low (–1 SD), the slope of state avoidance was significantly negative both for individuals low in trait avoidance (B = –7.26, *p* < .001) and high in trait avoidance (B = –7.09, *p* < .001). When interpersonal dysfunction was high (+1 SD), state avoidance again predicted lower interaction preference for both low trait avoidance (B = –5.93, *p* < .001) and high trait avoidance (B = –7.91, *p* < .001). Thus, although the three-way interaction reached significance, the substantive pattern indicates that state avoidance reliably reduced interaction preference for all individuals, with only modest variation in effect magnitude.

**Fig 5:**
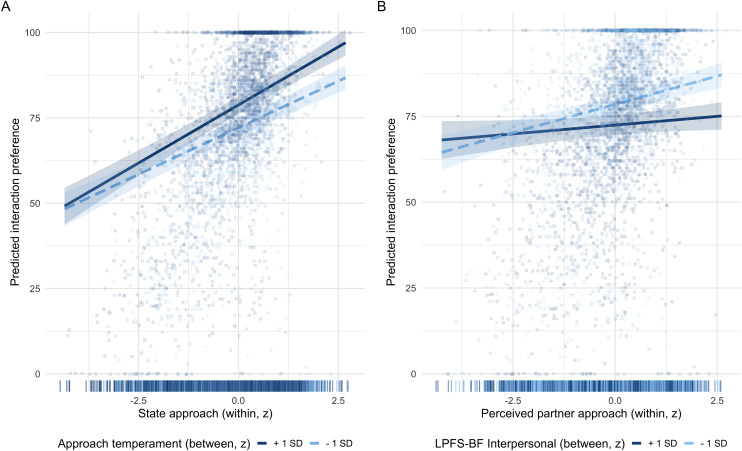
Interaction effects of personality traits, state motivations, and perceptions on social interaction preference. **(A)** Interaction between approach temperament and state approach motivation. **(B)** Interaction between perceived partner approach, and maladaptive interpersonal functioning (LPFS-BF Interpersonal). Shaded areas represent 95% confidence intervals.

In contrast, trait approach temperament increased interaction preferences particularly when individuals experienced higher state approach motivation (*B* = 0.67, 95% CI [0.07, 1.27], *p* = .029, Fig 5A). Simple-slopes analyses showed that state approach robustly predicted higher interaction preference at both low and high levels of trait approach. When trait approach was low (–1 SD), state approach was strongly associated with more positive interaction evaluations (B = 5.46, *p* < .001). When trait approach was high (+1 SD), this association was similarly strong (B = 6.80, *p* < .001). The Johnson–Neyman interval indicated that the effect of state approach was statistically significant across the entire observed range of trait approach. Thus, although the interaction reached significance, the substantive pattern reflects uniformly positive effects of state approach, with only modest strengthening among individuals high in trait approach temperament.

Perceiving interaction partners as approach-oriented improved interaction preference only for individuals with fewer deficits in interpersonal functioning (LPFS-BF Interpersonal, *B* = –1.17, 95% CI [–1.91, –0.42], *p* = .002, **Fig 5B**). Simple slopes revealed that when deficits in interpersonal functioning were low (–1 SD), perceiving partners as more approach-oriented strongly predicted higher interaction preference (B = 3.29, *p* < .001). When deficits in interpersonal functioning were high (+1 SD), this association was no longer statistically significant (B = 1.02, *p* = .064). These results suggest that perceiving others as approach-oriented boosts interaction experiences, but primarily for individuals without deficits in interpersonal functioning. No higher-order interactions reached significance (all values of *p* ≥ .096).

### Temporal dynamics of motivation and interaction preference

**Fig 6:**
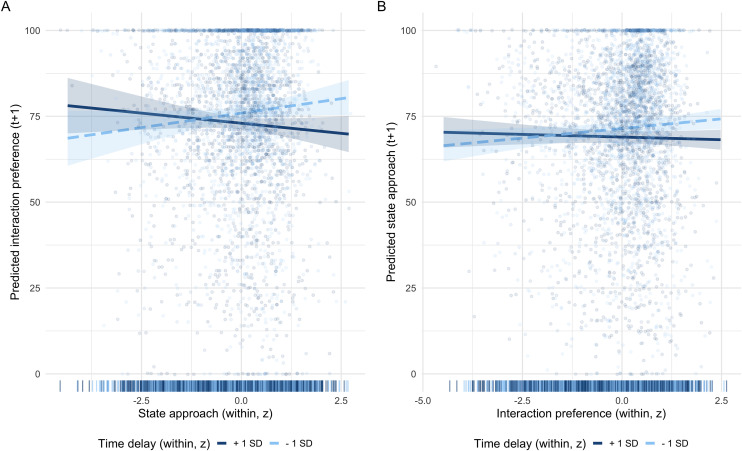
Lagged interaction effects of interaction preference and state motivations, depending on time delay. **(A)** Interaction between state approach and time delay on interaction preference at t + 1. **(B)** Interaction between interaction preference and time delay on state approach at t + 1. Shaded areas represent 95% confidence intervals.

Adding interaction preference (t) as an autoregressive predictor did not improve model fit (LRT: χ² [1] = 0.91, *p* = .341). Therefore, the simpler forward model was retained. Motivational states at t did not exhibit reliable main effects on liking at t + 1 (all *p*s ≥ .295), but a significant lagged state approach × Δtime interaction (B = −1.43, 95% CI [–2.71, –0.15], *p* = .029) indicated that short-term carryover weakened as inter-interaction intervals increased (**Fig 6A**). Simple-slopes analyses showed that when interactions occurred relatively soon after one another (–1 SD), lagged approach motivation showed a marginally positive but non-significant association with subsequent interaction preference (B = 1.68, *p* = .065). When interactions were spaced farther apart (+1 SD), the association was slightly negative but likewise non-significant (B = –1.18, *p* = .205). Together, these results indicate that approach-related carryover effects were weak and highly time-dependent, diminishing rapidly as the delay between interactions increased.

In the reverse models, higher interaction preference at t predicted stronger approach motivation at t + 1 only at short intervals (interaction preference × Δtime: B = −0.71, 95% CI [–1.36, –0.06], *p* = .032; **Fig 6B**). Simple slopes clarified this pattern: When interactions occurred in close temporal proximity (–1 SD), higher interaction preference at time t significantly predicted stronger state approach at t + 1 (B = 1.12, p = .018). In contrast, when interactions were spaced farther apart (+1 SD), there was no significant effect (B = –0.31, *p* = .505). No corresponding effects emerged for avoidance motivation (*p* = .153). Overall, these findings suggest that positive social experiences enhance subsequent approach motivation, but only when interactions follow one another relatively quickly, with the influence fading as delays increase.

Autoregressive stability of motivational states was strong (B = 1.92–2.04, *p*s < .001) but decayed with longer gaps (Δtime interactions: B = −1.13 to −1.51, *p*s < .001). These results indicate transient, time-sensitive coupling between motivation and interaction experience.

## Discussion

In this study, we present a novel, integrative approach to understanding social preferences by jointly modeling trait-level approach-avoidance temperament, motivational states, and perceived motivations of interaction partners in daily life. While prior research has examined these components separately, this is the first study to evaluate their unique and combined contributions using a high-resolution, two-week EMA design. Our findings demonstrate that both stable personality traits and states contribute to how individuals evaluate their social encounters. Combining trait, state, and perceived partner motivations provided the best explanatory power for predicting interaction preferences, supporting the notion that personality processes are inherently dynamic and context-dependent [6,35,36,38].

Consistent with our preregistered hypotheses, higher trait approach temperament, greater state approach motivation, and perceiving partners as more approach-oriented were associated with increased preference for social interactions. Conversely, avoidance states and perceiving partners as avoidant predicted lower interaction preference. These findings align with the approach-avoidance temperament [14,68], supporting the view that individuals’ motivational orientations, both dispositional and situational, play a critical role in shaping social experiences. Including state affect as control variables ensured that the associations between approach–avoidance motivation and interaction preference were not confounded by general affective valence. The fact that motivational effects remained robust when controlling for positive and negative affect indicates that they reflect directional motivational processes rather than mood alone [14]. Thus, approach and avoidance motivations appear to shape social evaluations above and beyond the influence of momentary affect.

Importantly, our results highlight that trait-level processes do not operate in isolation but interact with state-level motives and perceptions. The interaction between state approach and perceived partner approach, albeit small, suggests that when partners were already perceived as highly engaged, additional increases in one’s own approach motivation had a reduced impact on interaction preference. This finding reflects the dynamic interplay between self and other in social motivation, supporting theories of motivational interdependence [45].

Beyond these confirmatory findings, our exploratory analyses examined the role of deficits in interpersonal functioning. Deficits in interpersonal functioning were associated with overall lower interaction preference, but higher-order interactions involving interpersonal functioning showed only modest effects. Although some interaction terms reached statistical significance, simple-slopes analyses revealed that the underlying slopes were generally significant across all moderator levels, indicating limited evidence for qualitatively different processes as a function of deficits in interpersonal functioning. This pattern aligns with prior research showing that maladaptive personality functioning is broadly linked to heightened interpersonal reactivity, manifesting as stronger negative responses across many social contexts, yet often produces additive rather than sharply divergent interaction patterns [12,18,26,43,69]. For example, in our data, the three-way interaction involving deficits in interpersonal functioning, trait avoidance, and state avoidance reached significance, but state avoidance robustly predicted lower interaction preference at all levels of the moderators, consistent with findings that deficits in interpersonal functioning intensifies negative interpersonal experiences without fundamentally changing their direction [13].

A more differentiated pattern emerged for perceived partner approach. Perceiving partners as more approach-oriented predicted higher interaction preference particularly for individuals with low deficits in interpersonal functioning, whereas this association was attenuated, and no longer statistically significant, under higher deficits in interpersonal functioning. This pattern suggests that deficits in interpersonal functioning may limit the degree to which individuals benefit from positive social cues, consistent with interpersonal models of maladaptive functioning [12,26]. At the same time, the absence of strong divergences in simple-slopes patterns emphasizes that these moderation effects should be interpreted cautiously and require replication.

Taken together, these findings underscore the importance of integrating trait and state perspectives in personality research. While trait dispositions provide a baseline tendency, it is the interaction with momentary experiences and interpersonal perceptions that determines real-life social outcomes. Our study supports the Whole Trait Theory [6], emphasizing that personality expression is a product of both stable traits and situational variability. Moreover, although deficits in interpersonal functioning showed limited and nuanced moderation effects, its consistent negative association with interaction preference aligns with interpersonal theories of personality disorders, which posit that chronic dysfunction manifests through maladaptive patterns of perceiving and responding to others [13].

Beyond concurrent associations, we also explored short-term temporal dynamics between motivation and interaction preference using lagged multilevel models. Overall, these lagged effects were modest and highly time-dependent. In the forward direction, momentary motivational states did not show robust main effects on subsequent interaction preference once the time interval between interactions was taken into account; the interaction between prior state approach and the inter-interaction gap indicated that any carryover from approach motivation to later liking weakened as the delay between encounters increased. However, simple-slopes analyses showed that even at short lags, these effects were small and not reliably different from zero, suggesting that the influence of prior approach motivation on later evaluations is limited and dissipates quickly. In the reverse direction, higher interaction preference predicted stronger subsequent approach motivation only when interactions occurred in close temporal proximity, with no effect at longer intervals. This time-sensitive pattern is consistent with prior EMA research demonstrating that affective and interpersonal states typically show strong but short-lived autoregressive and cross-lagged effects that decay rapidly as temporal spacing increases [46,70–72]. Together, these findings suggest that motivation and interaction preference are coupled in a transient manner in daily life: positive interaction experiences can briefly boost approach motivation for the next encounter, but these effects diminish quickly as the time between interactions grows.

## Limitations and outlook

Several limitations should be acknowledged. First, while EMA offers ecological validity, self-reported measures of perceptions and motivations are subject to bias. Future studies could incorporate objective behavioral markers or partner-reported data (i.e., dyadic EMA) to provide more robust findings. Second, although our sample size provided sufficient power for detecting main and two-way effects, the study was not optimized for higher-order interactions (e.g., three- and four-way interaction effects). These complex terms require considerably larger samples to achieve stable estimation, and their interpretation should therefore be considered exploratory pending replication in future research. Third, participants in our study consisted predominantly of young adults, which may restrict the applicability of results to other age groups or clinical populations. Fourth, the generalizability of the present findings may be constrained by cultural factors. The functional meaning of avoidance motivation differs across cultural contexts, with avoidance-oriented goals being less detrimental – and sometimes even adaptive – in East Asian cultures compared to Western contexts [73,74]. Future work should examine whether the motivational dynamics observed here replicate across diverse cultural groups. Finally, our assessment of interaction preference relied on a single-item measure, which may limit reliability and reduce the precision with which momentary social preferences were captured.

Relatedly, our momentary affect indices were composed exclusively of high-arousal items (positive affect: enthusiastic, happy, determined; negative affect: worried, nervous, anxious), whereas low-arousal affective states (e.g., calm, relaxed, sad, or sluggish) were not assessed. Because low-arousal affect may be differentially relevant to avoidance motivation and to social withdrawal [75], the present findings speak primarily to high-arousal affective experience, and future work should sample the full arousal space to test whether these associations generalize.

Future studies should explore these dynamics in clinical populations with more extreme, i.e., maladaptive levels of personality traits to assess whether similar patterns emerge in more severe cases of deficits in interpersonal functioning. Additionally, leveraging longer EMA periods or experimental manipulations of social context could provide deeper insights into causal mechanisms. Another promising avenue involves examining emotion regulation strategies and their role in moderating approach-avoidance dynamics. Given the strong links between affect, motivation, and interpersonal functioning, integrating these domains could offer a more comprehensive understanding of social behavior in daily life.

## Conclusion

This study demonstrates that social interaction preferences are shaped by a complex interplay of personality traits, state-level motivations, and interpersonal perceptions. Although moderation effects involving deficits in interpersonal functioning were generally modest, they consistently predicted lower interaction preference and attenuated the benefits of perceiving partners as engaged. These findings highlight the value of dynamic, context-sensitive models of personality and underscore the potential for targeted interventions that enhance motivational flexibility and adaptive interpersonal perceptions in everyday social life. In terms of implications, the present results contribute on two levels. They support a process-oriented, integrative account of approach–avoidance dynamics that is likely to hold across populations: the finding that momentary states and partner perceptions outweigh stable traits in predicting interaction preference offers a transferable template for personality-in-context research and for interventions that target situational appraisals rather than dispositions alone. At the same time, because the sample was drawn from a single Western (German) context, the country-specific contribution is necessarily bounded; the meaning and adaptiveness of avoidance motivation are known to vary cross-culturally, so the magnitude and even the direction of some effects may differ in collectivistic or non-Western settings. We therefore frame our contribution as a generalizable framework that nonetheless requires country- and culture-specific calibration before its applied implications are extended beyond comparable Western samples.

## Supporting information

S1 File Supplementary Material. This file contains Figure S1 and Tables S1–S2, including extended baseline/EMA correlations and supplementary model comparisons.
(PDF)

## References

[1] Cacioppo JT, Cacioppo S. Social relationships and health: The toxic effects of perceived social isolation. Soc Personal Psychol Compass. 2014;8(2):58–72. doi: 10.1111/spc3.12087 24839458 PMC4021390

[2] Cohen S. Social relationships and health. Am Psychol. 2004;59(8):676–84. doi: 10.1037/0003-066X.59.8.676 15554821

[3] Kroencke L, Harari GM, Back MD, Wagner J. Well-being in social interactions: Examining personality-situation dynamics in face-to-face and computer-mediated communication. J Pers Soc Psychol. 2023;124(2):437–60. doi: 10.1037/pspp0000422 35834202

[4] Thoits PA. Mechanisms linking social ties and support to physical and mental health. J Health Soc Behav. 2011;52(2):145–61. doi: 10.1177/0022146510395592 21673143

[5] Fleeson W. Toward a structure- and process-integrated view of personality: traits as density distribution of states. J Pers Soc Psychol. 2001;80(6):1011–27. 11414368

[6] Fleeson W, Jayawickreme E. Whole Trait Theory. J Res Personal. 2015;56:82–92. doi: 10.1016/j.jrp.2014.10.009 PMC4472377 26097268

[7] Reis HT. Reinvigorating the concept of situation in social psychology. Pers Soc Psychol Rev. 2008;12(4):311–29. doi: 10.1177/1088868308321721 18812499

[8] Caspi A, Roberts BW, Shiner RL. Personality development: stability and change. Annu Rev Psychol. 2005;56:453–84. doi: 10.1146/annurev.psych.55.090902.141913 15709943

[9] Wrzus C, Roberts BW. Processes of Personality Development in Adulthood: The TESSERA Framework. Pers Soc Psychol Rev. 2017;21(3):253–77. doi: 10.1177/1088868316652279 27260302

[10] Pincus AL, Ansell EB. Interpersonal Theory of Personality. Handbook of Psychology. Wiley. 2003. 209–29. doi: 10.1002/0471264385.wei0509

[11] Hopwood CJ. Interpersonal Dynamics in Personality and Personality Disorders. Eur J Personal. 2018;32(5):499–524. doi: 10.1002/per.2155

[12] Hopwood CJ, Wright AGC, Ansell EB, Pincus AL. The interpersonal core of personality pathology. J Pers Disord. 2013;27(3):270–95. doi: 10.1521/pedi.2013.27.3.270 23735037 PMC3675800

[13] Wright AGC, Hopwood CJ, Skodol AE, Morey LC. Longitudinal validation of general and specific structural features of personality pathology. J Abnorm Psychol. 2016;125(8):1120–34. doi: 10.1037/abn0000165 27819472 PMC5119768

[14] Elliot AJ, Thrash TM. Approach-avoidance motivation in personality: approach and avoidance temperaments and goals. J Pers Soc Psychol. 2002;82(5):804–18. doi: 10.1037//0022-3514.82.5.804 12003479

[15] Elliot AJ. Approach and avoidance motivation. Handbook of approach and avoidance motivation. New York, NY, US: Psychology Press. 2008. p. 3–14.

[16] Gable SL. Approach and avoidance social motives and goals. J Pers. 2006;74(1):175–222. doi: 10.1111/j.1467-6494.2005.00373.x 16451230

[17] Kaurin A, Pilkonis PA, Wright AGC. Attachment manifestations in daily interpersonal interactions. Affect Sci. 2022;3(3):546–58. doi: 10.1007/s42761-022-00117-6 36381494 PMC9537404

[18] Vize CE, Ringwald WR, Scott LN, Kamarck TW, Pilkonis PA, Wright AGC. Evidence for a vicious socioemotional cycle of negative emotions and interpersonal conflict. J Consult Clin Psychol. 2024;92(8):479–92. doi: 10.1037/ccp0000891 39347785 PMC11837249

[19] Wright AGC, Hopwood CJ, Simms LJ. Daily Interpersonal and Affective Dynamics in Personality Disorder. J Pers Disord. 2015;29(4):503–25. doi: 10.1521/pedi.2015.29.4.503 26200849 PMC4511964

[20] Schafer M, Schiller D. Social avoidance can be quantified as navigation in abstract social space. Commun Psychol. 2025;3(1):51. doi: 10.1038/s44271-025-00215-8 40133401 PMC11936828

[21] Abrahams L, Rauthmann JF, De Fruyt F. Understanding person-situation dynamics at work: Effects of traits, states, and situation characteristics on teaching performance. Soc Psychol Personal Sci. 2025;16(5):572–84. doi: 10.1177/19485506241236812

[22] Reis HT, Lemay EP, Finkenauer C. Toward understanding understanding: The importance of feeling understood in relationships. Soc Personal Psychol Compass. 2017;11(3). doi: 10.1111/spc3.12308

[23] Weiß M, Gründahl M, Jachnik A, Hein G. Who Is Interacting With Whom? Assessing the Relationship Between Personality Traits and Preferences for Interaction Partners in Real Life. Collabra: Psychology. 2023;9(1). doi: 10.1525/collabra.91094

[24] Karney BR, Bradbury TN. The longitudinal course of marital quality and stability: a review of theory, method, and research. Psychol Bull. 1995;118(1):3–34. doi: 10.1037/0033-2909.118.1.3 7644604

[25] McNulty JK, Meltzer AL, Neff LA, Karney BR. How both partners’ individual differences, stress, and behavior predict change in relationship satisfaction: extending the VSA model. Proceedings of the National Academy of Sciences. 2021;118(27):e2101402118. doi: 10.1073/pnas.2101402118 PMC8271655 34183417

[26] Sadikaj G, Moskowitz DS, Russell JJ, Zuroff DC, Paris J. Quarrelsome behavior in borderline personality disorder: influence of behavioral and affective reactivity to perceptions of others. J Abnorm Psychol. 2013;122(1):195–207. doi: 10.1037/a0030871 23231460

[27] Halberstadt AL, Pincus AL, Mogle J, Ansell EB. Interpersonal Complementarity and Affect in Daily Life. Motiv Emot. 2023;47(2):270–81. doi: 10.1007/s11031-022-10003-0 38983372 PMC11233140

[28] Ringwald WR, Wright AGC. The Affiliative Role of Empathy in Everyday Interpersonal Interactions. Eur J Pers. 2021;35(2):197–211. doi: 10.1002/per.2286 34970022 PMC8716022

[29] Sadikaj G, Moskowitz DS, Zuroff DC. Intrapersonal variability in interpersonal perception in romantic relationships: Biases and accuracy. Journal of Research in Personality. 2017;69:67–77. doi: 10.1016/j.jrp.2016.06.011

[30] Cloutier J, Heatherton TF, Whalen PJ, Kelley WM. Are attractive people rewarding? Sex differences in the neural substrates of facial attractiveness. J Cogn Neurosci. 2008;20(6):941–51. doi: 10.1162/jocn.2008.20062 18211242 PMC3848031

[31] Langlois JH, Kalakanis L, Rubenstein AJ, Larson A, Hallam M, Smoot M. Maxims or myths of beauty? A meta-analytic and theoretical review. Psychol Bull. 2000;126(3):390–423. doi: 10.1037/0033-2909.126.3.390 10825783

[32] Voit M, Weiß M, Hewig J. The benefits of beauty – individual differences in the pro-attractiveness bias in social decision making. Current Psychology. 2021. doi: 10.1007/s12144-021-02366-3

[33] Aharon I, Etcoff N, Ariely D, Chabris CF, O’Connor E, Breiter HC. Beautiful faces have variable reward value: fMRI and behavioral evidence. Neuron. 2001;32(3):537–51. doi: 10.1016/s0896-6273(01)00491-3 11709163

[34] O’Doherty J, Winston J, Critchley H, Perrett D, Burt DM, Dolan RJ. Beauty in a smile: the role of medial orbitofrontal cortex in facial attractiveness. Neuropsychologia. 2003;41(2):147–55. doi: 10.1016/s0028-3932(02)00145-8 12459213

[35] Columbus S, Strandsbjerg CF. Personality states. Curr Opin Psychol. 2025;65:102099. doi: 10.1016/j.copsyc.2025.102099 40700981

[36] Fleeson W, Jayawickreme E. Getting from states to traits: Whole Trait Theory’s explanatory and developmental engine. Eur J Personal. 2025. doi: 10.1177/08902070251366709

[37] Kritzler S, Horstmann KT, Quintus M, Egloff B, Wrzus C, Luhmann M. Are state-trait fit and state-situation fit relevant for within-person dynamics of personality states?. J Pers Soc Psychol. 2024;127(4):880–900. doi: 10.1037/pspp0000519 39570691

[38] McCabe KO, Fleeson W. Are traits useful? Explaining trait manifestations as tools in the pursuit of goals. J Pers Soc Psychol. 2016;110(2):287–301. doi: 10.1037/a0039490 26280839 PMC4718867

[39] Shiffman S, Stone AA, Hufford MR. Ecological momentary assessment. Annu Rev Clin Psychol. 2008;4:1–32. doi: 10.1146/annurev.clinpsy.3.022806.091415 18509902

[40] Machell KA, Goodman FR, Kashdan TB. Experiential avoidance and well-being: a daily diary analysis. Cogn Emot. 2015;29(2):351–9. doi: 10.1080/02699931.2014.911143 24800802

[41] Updegraff JA, Gable SL, Taylor SE. What makes experiences satisfying? The interaction of approach-avoidance motivations and emotions in well-being. J Pers Soc Psychol. 2004;86(3):496–504. doi: 10.1037/0022-3514.86.3.496 15008652

[42] Sadikaj G, Moskowitz DS, Zuroff DC. Attachment-related affective dynamics: differential reactivity to others’ interpersonal behavior. J Pers Soc Psychol. 2011;100(5):905–17. doi: 10.1037/a0022875 21443369

[43] Vize CE, Kaurin A, Wright AGC. Personality pathology and momentary stress processes. Clin Psychol Sci. 2024;12(4):686–705. doi: 10.1177/21677026231192483 39119069 PMC11309262

[44] Wrzus C, Luong G, Wagner GG, Riediger M. Longitudinal coupling of momentary stress reactivity and trait neuroticism: Specificity of states, traits, and age period. J Pers Soc Psychol. 2021;121(3):691–706. doi: 10.1037/pspp0000308 34323531 PMC8763299

[45] Pusch S, Schönbrodt FD, Zygar-Hoffmann C, Hagemeyer B. Motivational interdependence in couple relationships. Front Psychol. 2022. doi: 10.3389/fpsyg.2022.827746 PMC9169053 35677131

[46] Wrzus C, Roos Y, Krämer MD, Schoedel R, Back MD, Richter D. Affiliation motive and social interactions in people’s daily life: A temporal processes approach using ecological momentary assessment and mobile sensing. J Pers Soc Psychol. 2025;129(2):341–62. doi: 10.1037/pspp0000555 40388128

[47] Zygar-Hoffmann C, Pusch S, Hagemeyer B, Schönbrodt FD. Motivated Behavior in Intimate Relationships: Comparing the Predictive Value of Motivational Variables. Soc Psychol Bull. 2020;15(2):2. doi: 10.32872/spb.2873

[48] Pickett CL, Gardner WL. The social monitoring system: Enhanced sensitivity to social cues as an adaptive response to social exclusion. The social outcast: Ostracism, social exclusion, rejection, and bullying. New York, NY, US: Psychology Press. 2005. p. 213–26.

[49] Jakubiak BK, Debrot A, Kim J, Impett EA. Approach and avoidance motives for touch are predicted by attachment and predict daily relationship well-being. J Soc Pers Relatsh. 2021;38(1):256–78. doi: 10.1177/0265407520961178

[50] Parrish EM, Chalker S, Cano M, Harvey PD, Taylor CT, Pinkham A, et al. Ecological Momentary Assessment of Social Approach and Avoidance Motivations in Serious Mental Illness: Connections to Suicidal Ideation and Symptoms. Arch Suicide Res. 2024;28(1):123–40. doi: 10.1080/13811118.2022.2137445 36377277 PMC10183051

[51] Strauss GP, Raugh IM, Luther L, Walker EF, Mittal VA. Temporal Interactions Between Social Motivation and Behavior In Daily Life Among Individuals at Clinical High-Risk for Psychosis. Schizophr Bull. 2023;49(5):1150–60. doi: 10.1093/schbul/sbad096 37467481 PMC10483454

[52] Kazak AE. Editorial: Journal article reporting standards. Am Psychol. 2018;73(1):1–2. doi: 10.1037/amp0000263 29345483

[53] Kleiman E. EMAtools: Data management tools for real-time monitoring/ecological momentary assessment data. 2017.

[54] Hepp J, Carpenter RW, Lane SP, Trull TJ. Momentary symptoms of borderline personality disorder as a product of trait personality and social context. Personal Disord. 2016;7(4):384–93. doi: 10.1037/per0000175 26901455 PMC4993692

[55] McMahon TP, Naragon-Gainey K. The multilevel structure of daily emotion-regulation-strategy use: An examination of within- and between-person associations in naturalistic settings. Clin Psychol Sci. 2019;7(2):321–39. doi: 10.1177/2167702618807408

[56] Uliaszek AA, Magurno A, Zedan S, Fournier MA. Emptiness, personality dysfunction, and emotion dysregulation: An experience sampling study. Personal Disord. 2025;16(5):395–402. doi: 10.1037/per0000737 40388124

[57] Adler NE, Epel ES, Castellazzo G, Ickovics JR. Relationship of subjective and objective social status with psychological and physiological functioning: preliminary data in healthy white women. Health Psychol. 2000;19(6):586–92. doi: 10.1037//0278-6133.19.6.586 11129362

[58] Paelecke M, Weiß M. German validation of the approach-avoidance temperament questionnaire. Eur J Psychol Assess. 2025. 10.1080/00223891.2024.2350466 PMC11582082 38776435

[59] Rassaby M, Rogers JM, Taylor CT. Validation of the Approach-Avoidance Temperament Questionnaire in Individuals with Anxiety and Depression. J Pers Assess. 2025;107(1):114–26. doi: 10.1080/00223891.2024.2350466 38776435 PMC11582082

[60] Spitzer C, Müller S, Kerber A, Hutsebaut J, Brähler E, Zimmermann J. The German Version of the Level of Personality Functioning Scale-Brief Form 2.0 (LPFS-BF): Latent Structure, Convergent Validity and Norm Values in the General Population. Psychother Psychosom Med Psychol. 2021;71(7):284–93. doi: 10.1055/a-1343-2396 33694153

[61] Bach B, Kerber A, Aluja A, Bastiaens T, Keeley JW, Claes L. International assessment of DSM-5 and ICD-11 personality disorder traits: toward a common nosology in DSM-5.1. Psychopathology. 2020;53(3–4):179–88. doi: 10.1159/000507589 32369820

[62] Elliot AJ, Gable SL, Mapes RR. Approach and avoidance motivation in the social domain. Pers Soc Psychol Bull. 2006;32(3):378–91. doi: 10.1177/0146167205282153 16455864

[63] Sadikaj G, Moskowitz DS. I hear but I don’t see you: Interacting over phone reduces the accuracy of perceiving affiliation in the other. Comput Hum Behav. 2018;89:140–7. doi: 10.1016/j.chb.2018.08.004

[64] Watson D, Clark LA, Tellegen A. Development and validation of brief measures of positive and negative affect: the PANAS scales. J Pers Soc Psychol. 1988;54(6):1063–70. doi: 10.1037//0022-3514.54.6.1063 3397865

[65] Bolger N, Laurenceau JP. Intensive Longitudinal Methods: An Introduction to Diary and Experience Sampling Research. Guilford Press. 2013.

[66] Burnham KP, Anderson DR. Multimodel inference: understanding AIC and BIC in model selection. Sociol Methods Res. 2004;33(2):261–304. doi: 10.1177/0049124104268644

[67] Nakagawa S, Johnson PCD, Schielzeth H. The coefficient of determination R2 and intra-class correlation coefficient from generalized linear mixed-effects models revisited and expanded. J R Soc Interface. 2017;14(134):20170213. doi: 10.1098/rsif.2017.0213 28904005 PMC5636267

[68] Elliot AJ, Thrash TM. Approach and avoidance temperament as basic dimensions of personality. J Pers. 2010;78(3):865–906. doi: 10.1111/j.1467-6494.2010.00636.x 20573129

[69] Sanchez-Lopez A. Ecological Momentary Assessments as a New Approach to Understand Differential Socio-Affective Features Characterizing Psychopathy Dimensions. Clinical Psychological Science. 2025;14(3):360–5. doi: 10.1177/21677026251338024

[70] Brose A, Schmiedek F, Lövdén M, Lindenberger U. Normal aging dampens the link between intrusive thoughts and negative affect in reaction to daily stressors. Psychol Aging. 2011;26(2):488–502. doi: 10.1037/a0022287 21480717

[71] Houben M, Van Den Noortgate W, Kuppens P. The relation between short-term emotion dynamics and psychological well-being: A meta-analysis. Psychol Bull. 2015;141(4):901–30. doi: 10.1037/a0038822 25822133

[72] Kuppens P, Oravecz Z, Tuerlinckx F. Feelings change: accounting for individual differences in the temporal dynamics of affect. J Pers Soc Psychol. 2010;99(6):1042–60. doi: 10.1037/a0020962 20853980

[73] Elliot AJ, Chirkov VI, Kim Y, Sheldon KM. A cross-cultural analysis of avoidance (relative to approach) personal goals. Psychol Sci. 2001;12(6):505–10. doi: 10.1111/1467-9280.00393 11760139

[74] Heine SJ, Lehman DR, Markus HR, Kitayama S. Is there a universal need for positive self-regard?. Psychol Rev. 1999;106(4):766–94. doi: 10.1037/0033-295X.106.4.766 10560328

[75] Adamis AM, Jessup SC, Cole DA, Olatunji BO. Ecological momentary assessment of the unique effects of trait worry on daily negative emotionality: does arousal matter?. Cogn Emot. 2025;39(3):714–21. doi: 10.1080/02699931.2024.2386124 39093304

