# Supplementary material for: When traits meet the moment: State and perceived partner motivations interact with approach-avoidance temperament to predict social preferences in daily life
Source: PLoS One. 2026 Aug 5;21(8):e0353754. doi: 10.1371/journal.pone.0353754 (PMC13440843; doi:10.1371/journal.pone.0353754)
Supplement: Supplementary file 1 [file pone.0353754.s001.pdf]

Supplementary Material

When traits meet the moment: State and perceived partner motivations interact with approach-avoidance temperament to predict social preferences in daily life

Broader personality correlates of motivational dispositions

To provide a broader view of personality–motivation associations, we extended the between-person correlation analysis to include Personality Inventory for DSM-5 (PID-5) domains and Friendship Goals. As shown in **Figure S1**, the five PID domains were moderately to strongly intercorrelated (rs = .23–.51, all ps < .001) and aligned with overall deficits in interpersonal functioning (LPFS-BF Interpersonal; rs = .26–.57). Higher PID Negative Affectivity was most strongly associated with higher trait avoidance temperament (r = .74) and Detachment most strongly with lower trait approach temperament (r = –.37), indicating consistent convergence with motivational dispositions. PID Disinhibition, Anankastia, and Psychoticism likewise correlated positively with avoidance temperament (rs = .32–.40, all ps < .001) but showed negligible relations with approach temperament.

For friendship goals, approach-oriented goals showed a small positive association with interaction preference (r = .21, p = .013), whereas avoidance-oriented friendship goals exhibited no significant correlation with any state variable from EMA. Overall, the extended correlational pattern suggests that maladaptive personality traits covary primarily with avoidance-oriented dispositions and deficits in interpersonal functioning, whereas approach-related friendship goals relate modestly to positive interaction preferences.

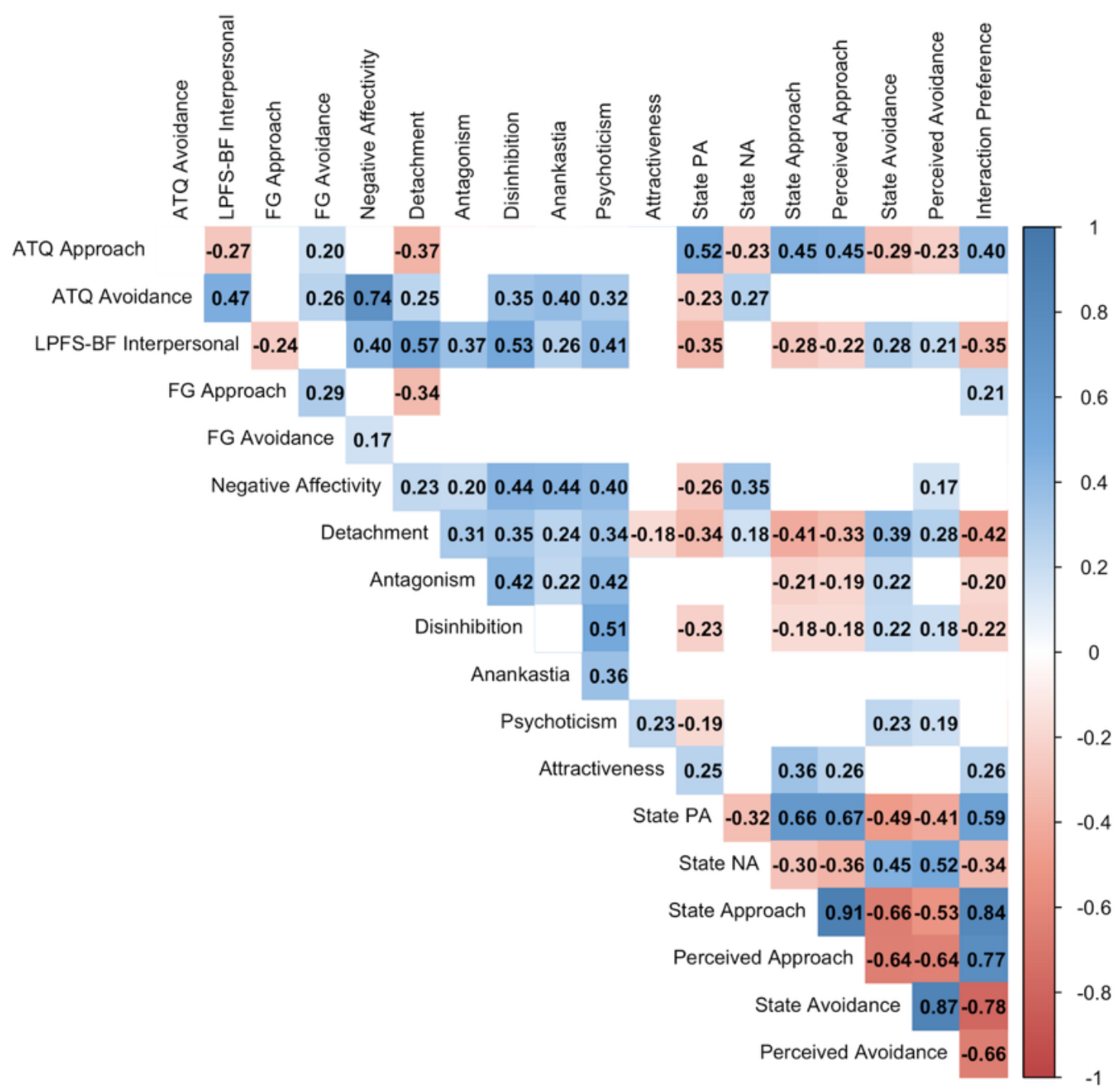

Figure S1. Between-person correlations among extended baseline and EMA variables.

Stability of motivational dispositions across trait and state levels

To test whether aggregated motivational states could serve as trait-equivalent indicators of stable between-person differences, we compared models that used baseline trait measures (ATQ Approach and ATQ Avoidance) with models that used aggregated mean levels of EMA state approach and avoidance (**Table S1**). Both sets of predictors were entered at the between-person level (i.e., grand-mean centered per participant).

The model including aggregated EMA states instead of approach avoidance temperament showed a substantially better fit than the baseline trait model (M1; AIC = 34123 vs. 34321; ΔAIC = −198; $\chi^2(2) = 197.96$, $p < .001$). When both operationalizations were entered simultaneously, only the aggregated EMA means remained significant ($\chi^2(2) = 198.21$, $p < .001$ for added states; $\chi^2(2) = 0.26$, $p = .890$ for added traits), indicating that stable individual differences in approach–avoidance motivation were better captured by aggregated EMA states than by baseline trait measures.

Conceptually, these findings align with Whole Trait Theory (Fleeson, 2001), which posits that stable traits correspond to the density distributions of states over time. Hence, aggregated EMA measures provide convergent evidence for the stability of approach–avoidance motivation across contexts. We retained baseline traits in the preregistered main analyses to ensure conceptual clarity and comparability with prior research, while noting that all effects generalize across trait operationalizations.

Table S1. Comparison of Baseline Trait and Aggregated EMA-State Models.

Model	AIC	ΔAIC	BIC	ΔBIC	χ^2 (df = 2)	p	R^2 (marg.)	R^2 (cond.)
M1 – Traits only	34321	—	34372	—	—	—	0.33	0.48
M1 – Aggregated states only	34123	−198	34174	−198	197.96	< .001	0.46	0.48
M1 – Traits + aggregated states	34127	−194	34190	−182	—	—	0.46	0.48

Note. ΔAIC and χ^2 statistics indicate relative model improvement. Models estimated with random intercepts for participants.

Disentangling motivation from affective valence

To evaluate whether controlling for affective valence influenced the motivational effects, we re-estimated the state-only (M2), perceived-state (M3), and combined (M4) models without PANAS predictors and compared them using likelihood-ratio tests (**Table S2**). Including positive and negative affect as control variables significantly improved model fit (*p*s < .001) but did not alter the magnitude or direction of motivational predictors. This indicates that approach–avoidance associations with interaction preference are not reducible to affective valence, supporting their interpretation as directional motivational processes rather than mood-related artifacts.

Table S2. Model Comparisons Including vs. Excluding PANAS Affect Controls.

Model	AIC	ΔAIC	BIC	ΔBIC	χ^2(df = 2)	p	R^2 (marg.)	R^2 (cond.)
M2 excl. PANAS	32474	—	32512	—	—	—	0.48	0.68
**M2 incl. PANAS**	**32394**	**-80**	**32444**	**-68**	84.1	< .001	**0.49**	**0.69**
M3 excl. PANAS	34084	—	34122	—	—	—	0.32	0.52
**M3 incl. PANAS**	**33516**	**-568**	**33566**	**-556**	82.7	< .001	**0.39**	**0.58**
M4 excl. PANAS	32360	—	32474	—	—	—	0.53	0.69
**M4 incl. PANAS**	**32293**	**-67**	**32419**	**-55**	84.1	< .001	**0.53**	**0.70**

Note. PANAS = Positive and Negative Affect Schedule. ΔAIC = difference relative to the corresponding model without affect controls. Models estimated with random intercepts for participants.

Cross-level interactions between trait temperament and state motivation

To examine whether individuals high versus low in trait approach or avoidance temperament differed in how momentary motivational states shaped their interaction evaluations, we estimated a cross-level model including all trait × state × perceived-state interactions for both motivational systems (approach and avoidance). Model fit did not improve statistically relative to the combined model (M4) ($\chi^2(6) = 12.01$, $p = .062$), indicating that these additional higher-order interactions did not increase explanatory power.

Across predictors, several main effects remained robustly significant: higher trait approach predicted higher interaction preference (B = 4.38, 95% CI [2.68, 6.09], $p < .001$). Likewise, state approach (B = 6.20, 95% CI [5.39, 7.02], $p < .001$) and perceived partner approach (B = 2.04, 95% CI [1.30, 2.79], $p < .001$) were both strong positive predictors. Higher state positive affect (B = 2.17, 95% CI [1.59, 2.75], $p < .001$) and greater partner attractiveness (B = 1.05, 95% CI [0.62, 1.48], $p < .001$) also predicted more favorable interaction evaluations.

In contrast, avoidance-related states were associated with lower interaction preference: state avoidance (B = –7.44, 95% CI [–8.13, –6.75], $p < .001$) and perceived partner avoidance (B = –0.78, 95% CI [–1.47, –0.10], $p = .024$) both exerted significant negative effects.

Two interaction effects reached significance. First, a negative interaction between state approach and perceived partner approach (B = –0.46, 95% CI [–0.84, –0.08], $p = .017$) replicated the diminishing-returns pattern observed in the confirmatory model. Second, avoidance temperament showed a cross-motivation interaction with perceived partner approach (B = –0.96, 95% CI [–1.68, –0.24], $p = .009$), indicating that individuals with higher avoidance temperament evaluated interactions less positively when perceiving their partners as highly approach-motivated.

All remaining interaction terms, including all three-way interactions, were non-significant (all *p*s ≥ .100), suggesting that the situational effects of motivational states were largely comparable across individuals regardless of their broader motivational dispositions.
