# Supplementary material for: When traits meet the moment: State and perceived partner motivations interact with approach-avoidance temperament to predict social preferences in daily life
Source: PLoS One. 2026 Aug 5;21(8):e0353754. doi: 10.1371/journal.pone.0353754 (PMC13440843; doi:10.1371/journal.pone.0353754)
Supplement: Supplementary file 2 [file pone.0353754.s003.docx]

Dear Dr. Tovmasyan,

Thank you very much for the opportunity to revise our manuscript, and for the constructive and detailed feedback provided by you and Reviewer #1. We have worked carefully to address every point regarding presentation, clarity, and completeness. Editor and reviewer comments are reproduced in bold type, followed by our responses in italics. In the revised manuscript, all changes are marked using “Track Changes”.

**Responses to Editor’s comments:**

**First, the introduction could be clearer in the way it synthesises existing models. I would suggest including a diagram that integrates variables from different models you have included. Further, a bit more detail on each model will assist the readability. It would be helpful to include concrete examples, e.g. “The continuity principle highlights that individuals actively create environments consistent with their traits (*include example*), whereas TESSERA specify (*should be specifies*) micro-level feedback loops through which repeated state–situation couplings consolidate or change traits (*include an example of what this looks like in practice*).” Similarly, more detail is needed on the agency-communion dimension.**

*Response: We thank you for this helpful suggestion and have revised the introduction accordingly:*

- *We added a new integrative figure (new* ***Figure 1****) that places the variables from the reviewed frameworks into an overview, distinguishing the trait, state, and perceived-partner levels, the moderating role of interpersonal functioning, the predicted paths to interaction preference, and the feedback loop in the sense of TESSERA.*
- *We corrected “specify” to “specifies.”*
- *We added concrete examples for both the continuity principle and the TESSERA sequence.*
- *We expanded the description of the agency–communion (interpersonal circumplex) dimension and linked it explicitly to approach–avoidance motivation.*

*New Figure 1:*

***Figure 1. An integrative approach–avoidance model of social interaction preference.*** *Momentary interaction preference is predicted by three levels: stable traits (approach/avoidance temperament; ATQ), within-person states (state approach/avoidance), and partner appraisals (perceived partner approach/avoidance). Traits predispose states, which couple with partner perceptions during an encounter. Interpersonal functioning (LPFS-BF Interpersonal) moderates these paths, especially avoidance-related effects. Solid colored arrows = predictive paths estimated in the multilevel models; grey dashed arrows = moderation; the orange dashed arrow = the theoretically expected TESSERA feedback loop (consolidation of states into traits over time), which is conceptual and not tested here.*

*The following paragraphs have been added to the introduction:*

*“The continuity principle highlights that individuals actively create environments consistent with their traits (e.g., a sociable person who repeatedly seeks out group activities and, in doing so, reinforces their own outgoing disposition), whereas TESSERA specifies micro-level feedback loops through which repeated state–situation couplings consolidate or change traits (e.g., a socially anxious person who, on entering a gathering, anticipates rejection, feels tense, and withdraws; across many such episodes these repeated state expressions gradually consolidate into a more avoidant trait pattern).”*

*“On this circumplex, the agency dimension captures the extent to which behavior is dominant versus submissive, whereas the communion dimension captures the extent to which it is warm versus cold or hostile. Approach motivation maps most closely onto agentic and communal (i.e., engaged, warm) behavior, whereas avoidance motivation maps onto cold and withdrawn behavior, which is why the agency–communion framework provides a useful descriptive language for the motivational states examined here.”*

**Second, as Reviewer 1 has pointed out, the literature review relies on papers published prior to 2024. Is there any recent literature that you could include? This is particularly important where you say “advances in personality and social psychology emphasize the need to integrate state-level variability with trait-level perspectives to better predict behaviour and subjective experiences (Fleeson, 2001; McCabe & Fleeson, 2016)”, yet papers from 2001 and 2016 would not be considered to be “advances” in 2026.**

*Response: We agree. We have reframed the flagged sentence so Fleeson, 2001 and McCabe & Fleeson, 2016 are presented as foundational rather than as recent “advances,” and we have integrated recent (2024–2026) literature at the relevant points in the introduction and discussion:*

*“In consequence, a well-established line of work in personality and social psychology has emphasized the need to integrate state-level variability with trait-level perspectives…”*

*Added references:*

Abrahams, L., Rauthmann, J. F., & De Fruyt, F. (2025). Understanding Person-Situation Dynamics at Work: Effects of Traits, States, and Situation Characteristics on Teaching Performance. *Social Psychological and Personality Science*, *16*(5), 572–584. <https://doi.org/10.1177/19485506241236812>

Adamis, A. M., Jessup, S. C., Cole, D. A., & Olatunji, B. O. (2025). Ecological momentary assessment of the unique effects of trait worry on daily negative emotionality: Does arousal matter? *Cognition & Emotion*, *39*(3), 714–721. <https://doi.org/10.1080/02699931.2024.2386124>

Columbus, S., & Strandsbjerg, C. F. (2025). Personality states. *Current Opinion in Psychology*, *65*, 102099. <https://doi.org/10.1016/j.copsyc.2025.102099>

Fleeson, W., & Jayawickreme, E. (2025). Getting from states to traits: Whole Trait Theory’s explanatory and developmental engine. *European Journal of Personality*, 08902070251366709. <https://doi.org/10.1177/08902070251366709>

Kritzler, S., Horstmann, K. T., Quintus, M., Egloff, B., Wrzus, C., & Luhmann, M. (2024). Are state-trait fit and state-situation fit relevant for within-person dynamics of personality states? *Journal of Personality and Social Psychology*, *127*(4), 880–900. <https://doi.org/10.1037/pspp0000519>

Rassaby, M., Rogers, J. M., & Taylor, C. T. (2025). Validation of the Approach-Avoidance Temperament Questionnaire in Individuals with Anxiety and Depression. *Journal of Personality Assessment*, *107*(1), 114–126. <https://doi.org/10.1080/00223891.2024.2350466>

Sanchez-Lopez, A. (2026). Ecological Momentary Assessments as a New Approach to Understand Differential Socio-Affective Features Characterizing Psychopathy Dimensions. *Clinical Psychological Science*, *14*(3), 360–365. <https://doi.org/10.1177/21677026251338024>

Schafer, M., & Schiller, D. (2025). Social avoidance can be quantified as navigation in abstract social space. *Communications Psychology*, *3*, 51. <https://doi.org/10.1038/s44271-025-00215-8>

Wrzus, C., Roos, Y., Krämer, M. D., Schoedel, R., Back, M. D., & Richter, D. (2025). Affiliation motive and social interactions in people’s daily life: A temporal processes approach using ecological momentary assessment and mobile sensing. *Journal of Personality and Social Psychology*, *129*(2), 341–362. <https://doi.org/10.1037/pspp0000555>

**Third, a minor point is that I would suggest including the name of the app you have used earlier on, on page 6.**

*Response: The app name (m-Path) is now first mentioned in the Participants section.*

**Fourth, when describing participants’ genders, please change “diverse” to “other” and “did not want to indicate” to “did not wish to indicate”.**

*Response: Done as suggested.*

**Fifth, please specify whether participants were presented with a participant information sheet and whether they were debriefed after the study.**

*Response: We have added a statement to the Methods (Participants) confirming that an information overview was provided before consent and that participants were debriefed at the end of the EMA period:*

*“Before providing consent, all participants were given a participant information overview describing the purpose, procedure, data handling, and the voluntary and confidential nature of participation, and they were debriefed about the aims of the study at the end of the EMA period.”*

**Sixth, it is not clear how long participants were given to answer the prompt. Did you discard late answers, or were participants not able to answer after some time?**

*Response: We have added a sentence to the Procedure clarifying the response window:*

*“Reminder notifications were sent 30 minutes after each prompt, and participants had a two-hour response window, after which the survey expired.“*

**Seventh, I was not able to locate supplementary materials with descriptive correlations.**

*Response: We apologize that the Supplementary Material was not successfully uploaded with the original submission; it is now attached to this revision. The descriptive correlations are reported in Figure S1 (a full correlation matrix of all baseline and EMA variables), complementing the focal correlations shown in Figure 2 (former Figure 1) of the main text.*

**Eighth, the inclusion of physical attractiveness of the interaction partner is not referenced in the introduction. Please provide a theoretical justification for including this variable.**

*Response: We have added a theoretical justification to the introduction, framing perceived partner attractiveness as a positively valenced reward cue within the approach–avoidance framework, and clarifying that it is included as a perception-level covariate so that motivational effects are not confounded with this basic reward signal.*

*We have added the following to the introduction:*

*“Among such partner cues, the perceived physical attractiveness of an interaction partner is a particularly salient reward signal that has been linked to heightened approach motivation (e.g., 30) and more positive social evaluations (31,32). From an approach–avoidance perspective, attractiveness functions as a positively valenced incentive that can amplify approach-related responding independently of a partner’s perceived motivation (33,34). We therefore included perceived partner attractiveness as a perception-level covariate in all models to ensure that the effects of motivational appraisals were not confounded with this basic reward cue.“*

**Ninth, it should be acknowledged in the limitations that you have only used high arousal affect items, and low arousal is not acknowledged in the study.**

*Response: We fully agree. We have added this acknowledgement to the Limitations section.*

*“Relatedly, our momentary affect indices were composed exclusively of high-arousal items (positive affect: enthusiastic, happy, determined; negative affect: worried, nervous, anxious), whereas low-arousal affective states (e.g., calm, relaxed, sad, or sluggish) were not assessed. Because low-arousal affect may be differentially relevant to avoidance motivation and to social withdrawal (75), the present findings speak primarily to high-arousal affective experience, and future work should sample the full arousal space to test whether these associations generalize.“*

**Tenth, please include each reliability score in the methods section where you describe the measures, rather than in results.**

*Response: We have moved the reliability estimates into the Methods (Measures) section, reporting them where each measure is introduced. They remain visible in Table 1 for completeness.*

**Minor – p. 25, line 620 – space is missing.**

*Response: Corrected. We also re-read the manuscript for similar spacing issues.*

**Response to Reviewer 1:**

**Reviewer #1: I am impressed by the author's work entitled "When traits meet the moment: State and perceived partner motivations interact with approach-avoidance temperament to predict social preferences in daily life". The authors did a great job in the manuscript especially articulation the gap and writing in an academic tone, however i have few suggestion for authors to improve some remaining parts of the manuscript.**

*Response: Thank you for the positive evaluation, your insightful feedback and detailed suggestions. Your comments have greatly helped us refine our manuscript and clarify our key findings.*

**1. My first suggestion to authors is to add and cite recent relevant literature from the 2026/2025/2024.**

*Response: We agree and have integrated recent (2024-2026) literature in the introduction and discussion (see Editor Comment 2).*

*The following references have been added:*

Abrahams, L., Rauthmann, J. F., & De Fruyt, F. (2025). Understanding Person-Situation Dynamics at Work: Effects of Traits, States, and Situation Characteristics on Teaching Performance. *Social Psychological and Personality Science*, *16*(5), 572–584. <https://doi.org/10.1177/19485506241236812>

Adamis, A. M., Jessup, S. C., Cole, D. A., & Olatunji, B. O. (2025). Ecological momentary assessment of the unique effects of trait worry on daily negative emotionality: Does arousal matter? *Cognition & Emotion*, *39*(3), 714–721. <https://doi.org/10.1080/02699931.2024.2386124>

Columbus, S., & Strandsbjerg, C. F. (2025). Personality states. *Current Opinion in Psychology*, *65*, 102099. <https://doi.org/10.1016/j.copsyc.2025.102099>

Fleeson, W., & Jayawickreme, E. (2025). Getting from states to traits: Whole Trait Theory’s explanatory and developmental engine. *European Journal of Personality*, 08902070251366709. <https://doi.org/10.1177/08902070251366709>

Kritzler, S., Horstmann, K. T., Quintus, M., Egloff, B., Wrzus, C., & Luhmann, M. (2024). Are state-trait fit and state-situation fit relevant for within-person dynamics of personality states? *Journal of Personality and Social Psychology*, *127*(4), 880–900. <https://doi.org/10.1037/pspp0000519>

Rassaby, M., Rogers, J. M., & Taylor, C. T. (2025). Validation of the Approach-Avoidance Temperament Questionnaire in Individuals with Anxiety and Depression. *Journal of Personality Assessment*, *107*(1), 114–126. <https://doi.org/10.1080/00223891.2024.2350466>

Sanchez-Lopez, A. (2026). Ecological Momentary Assessments as a New Approach to Understand Differential Socio-Affective Features Characterizing Psychopathy Dimensions. *Clinical Psychological Science*, *14*(3), 360–365. <https://doi.org/10.1177/21677026251338024>

Schafer, M., & Schiller, D. (2025). Social avoidance can be quantified as navigation in abstract social space. *Communications Psychology*, *3*, 51. <https://doi.org/10.1038/s44271-025-00215-8>

Wrzus, C., Roos, Y., Krämer, M. D., Schoedel, R., Back, M. D., & Richter, D. (2025). Affiliation motive and social interactions in people’s daily life: A temporal processes approach using ecological momentary assessment and mobile sensing. *Journal of Personality and Social Psychology*, *129*(2), 341–362. <https://doi.org/10.1037/pspp0000555>

**2. Secondly, i advised authors to shorten the long sentences in the manuscript, as it will enhance the readers interest.**

*Response: We have shortened and clarified several long sentences. For example, the previous sentence describing excluded interactions was both long and grammatically incomplete and has been rewritten:*

*Before: “Our study plan targeted recent real-life social interactions, which is why 1969 interactions more than 60 minutes ago 780 interactions took place virtually were not considered.”*

*After: “Because the study targeted recent, in-person social interactions, we did not consider 1,969 interactions that had occurred more than 60 minutes before the prompt or 780 interactions that had taken place virtually.”*

**3. Third suggestion is to critically synthesise the theory used in the study.**

*Response: The revised introduction now synthesizes the frameworks more critically and concretely. We have added the new integrative Figure 1, provided worked examples for the continuity principle and TESSERA, expanded the agency–communion description, and made explicit how the levels relate (traits predispose, states fluctuate in context, partner perceptions shape meaning, and interpersonal functioning moderates avoidance-related effects). Together these changes make the theoretical integration, rather than a list of models, the organizing thread of the introduction.*

*New Figure 1:*

***Figure 1. An integrative approach–avoidance model of social interaction preference.*** *Momentary interaction preference is predicted by three levels: stable traits (approach/avoidance temperament; ATQ), within-person states (state approach/avoidance), and partner appraisals (perceived partner approach/avoidance). Traits predispose states, which couple with partner perceptions during an encounter. Interpersonal functioning (LPFS-BF Interpersonal) moderates these paths, especially avoidance-related effects. Solid colored arrows = predictive paths estimated in the multilevel models; grey dashed arrows = moderation; the orange dashed arrow = the theoretically expected TESSERA feedback loop (consolidation of states into traits over time), which is conceptual and not tested here.*

**4. Fourth, The implications section should be revised, and it should be written in a comparative way of global and country based contributions.**

**thats all from my side i wish good luck to authors.**

*Response: We have revised the closing implications to distinguish a globally generalizable contribution (a transferable, process-oriented approach–avoidance framework) from the bounded, country-specific contribution of a single Western (German) sample, noting that the meaning and adaptiveness of avoidance motivation vary cross-culturally.*

*“In terms of implications, the present results contribute on two levels. They support a process-oriented, integrative account of approach–avoidance dynamics that is likely to hold across populations: the finding that momentary states and partner perceptions outweigh stable traits in predicting interaction preference offers a transferable template for personality-in-context research and for interventions that target situational appraisals rather than dispositions alone. At the same time, because the sample was drawn from a single Western (German) context, the country-specific contribution is necessarily bounded; the meaning and adaptiveness of avoidance motivation are known to vary cross-culturally, so the magnitude and even the direction of some effects may differ in collectivistic or non-Western settings. We therefore frame our contribution as a generalizable framework that nonetheless requires country- and culture-specific calibration before its applied implications are extended beyond comparable Western samples.“*
